# Biomechanical study of femoral neck system for young patients with nonanatomically reduced femoral neck fractures: a finite element

**DOI:** 10.1186/s12891-022-06124-9

**Published:** 2023-01-21

**Authors:** Zhirong Fan, Ping Chen, Xiubing Yu, Xing Li, Haitao Su, Haiyun Chen, Bing Yang, Ji Qi, Haizhou Wang

**Affiliations:** Guangdong Provincial Hospital of Traditional Chinese Medicine, Guangzhou University of Chinese Medicine, 510006 Guangzhou, China

**Keywords:** Femoral neck fracture, Femoral neck system, FNS, Positive buttress, Negative buttress, Finite element analysis

## Abstract

**Background:**

A consensus regarding the optimal approach for treating femoral neck fractures is lacking. We aimed to investigate the biomechanical outcomes of Femoral Neck System (FNS) internal fixation components in the treatment of nonanatomically reduced femoral neck fractures.

**Method:**

We constructed two types of femoral neck fractures of the Pauwels classification with angles of 30° and 50°, and three models of anatomic reduction, positive buttress reduction and negative buttress reduction were constructed. Subgroups of 1 to 4 mm were divided according to the distance of displacement in the positive buttress reduction and negative buttress reduction models. The von Mises stress and displacements of the femur and FNS internal fixation components were measured for each fracture group under 2100-N axial loads.

**Results:**

When the Pauwels angle was 30°, the positive 1-mm and 2-mm models had lower FNS stress than the negative buttress model. The positive 3- and 4-mm models showed FNS stress similar to that of the negative buttress model. But the four positive buttress models had similar stresses on the femur as the negative buttress model. When the Pauwels angle was 50°, the four positive buttress models had higher FNS stress than the negative buttress model. Three positive buttress models (2 mm, 3 and 4 mm) resulted in lower stress of the femur than the negative buttress model, though the 1-mm model did not. When the Pauwels angle was 30°, the positive buttress model had a lower displacement of the FNS than the negative buttress model and a similar displacement of the femur with the negative buttress model. When the Pauwels angle was 50°, the positive buttress model had a higher displacement of the FNS and femur than the negative buttress model. Our study also showed that the von Mises stress and displacement of the internal fixation and the femur increased as the fracture angle increased.

**Conclusion:**

From the perspective of biomechanics, when the Pauwels angle was 30°, positive buttress was more stable to negative buttress. However, when the Pauwels angle was 50°, this advantage weakens. In our opinion, the clinical efficacy of FNS internal fixation with positive buttress may be related to the fracture angle, neck-shaft angle and alignment in the lateral view. This result needs verification in further clinical studies.

## Introduction

Hip fracture is a common type of trauma. High-energy violence injuries are common in young patients, whereas osteoporotic fractures are seen in elderly patients. It is estimated that the rate of hip fracture will increase to 2.6 million by 2025 and 4.5 million by 2050 [1, 2]. Femoral neck fractures account for approximately half of all hip fractures [3], resulting in considerable socioeconomic burdens and medical challenges. Overall, hip fracture has high morbidity and mortality.

Treatment options for femoral neck fracture include internal fixation and artificial joint replacement. Patients under the age of 60 are considered to benefit from internal fixation, but those over the age of 80 have better outcomes with primary total hip arthroplasty [4]. Currently, internal fixation remains the gold standard for the treatment of femoral neck fractures in the young and nondisplaced femoral neck fractures in the elderly. Two major complications after surgical internal fixation of femoral neck fractures are avascular necrosis of the femoral head (AVN) and nonunion, which have been reported by many researchers [5–7]. The mainstream surgical interventions for femoral neck fracture in younger patients are cannulated screw fixation and dynamic hip screw (DHS) fixation [8–10]. However, the optimal internal fixation technique for unstable femoral neck fracture remains controversial. Consequently, clinicians have explored the next generation of effective fixation implants [11]. Femoral Neck System (FNS) is a novel device that was recently suggested by K. Stoffel to address femoral neck fracture [12]. Our previous research [13] also found that FNS is superior to cannulated screws in the treatment of anatomically reduced femoral neck fractures in terms of biomechanical stability. However, the efficacy of FNS is still controversial, so further investigation is needed.

Anatomical reduction is believed to be a critical factor in promoting femoral neck fracture healing and avoiding complications [14]. “Anatomical reduction” has never been challenged and questioned, and no alternatives have been proposed. Nevertheless, there are still many refractory femoral neck fractures that cannot be anatomically reduced by closed traction reduction, and repeated traction reduction will damage the remaining blood supply, thereby affecting fracture healing and the blood supply to the femoral head [15]. To address this problem, Gotfried et al. [16] proposed the technique of Gotfried reduction of unstable sub-cephalic femoral neck fracture in 2013, which is the so-called positive buttress for femoral neck fracture. Positive buttress refers to the distal femoral neck fragment located medially to the lower-medial edge of the proximal fracture fragment; negative buttress is opposite to the displacement direction. This is a new option for the treatment of refractory femoral neck fracture, and it has been accepted by many scholars. Wang et al [15] found that compared with negative buttress for femoral neck fracture, positive buttress can provide better biomechanical stability using inverted cannulated screws. To the best of our knowledge, however, there are no studies on the treatment of femoral neck fracture using FNS internal fixation under nonanatomical reduction for the treatment of femoral neck fracture. Therefore, based on our previous research [13], we continued to study the biomechanics of FNS stabilization under nonanatomical conditions of femoral neck fracture. The purpose of our study was to explore the stability of FNS under nonanatomical reduction in the treatment of femoral neck fracture. We hypothesized that positive buttress reduction is better than negative buttress reduction but that it varies with the fracture angle.

## Materials and methods

### Three-dimensional modeling of the femoral neck fracture

**Fig. 1:**
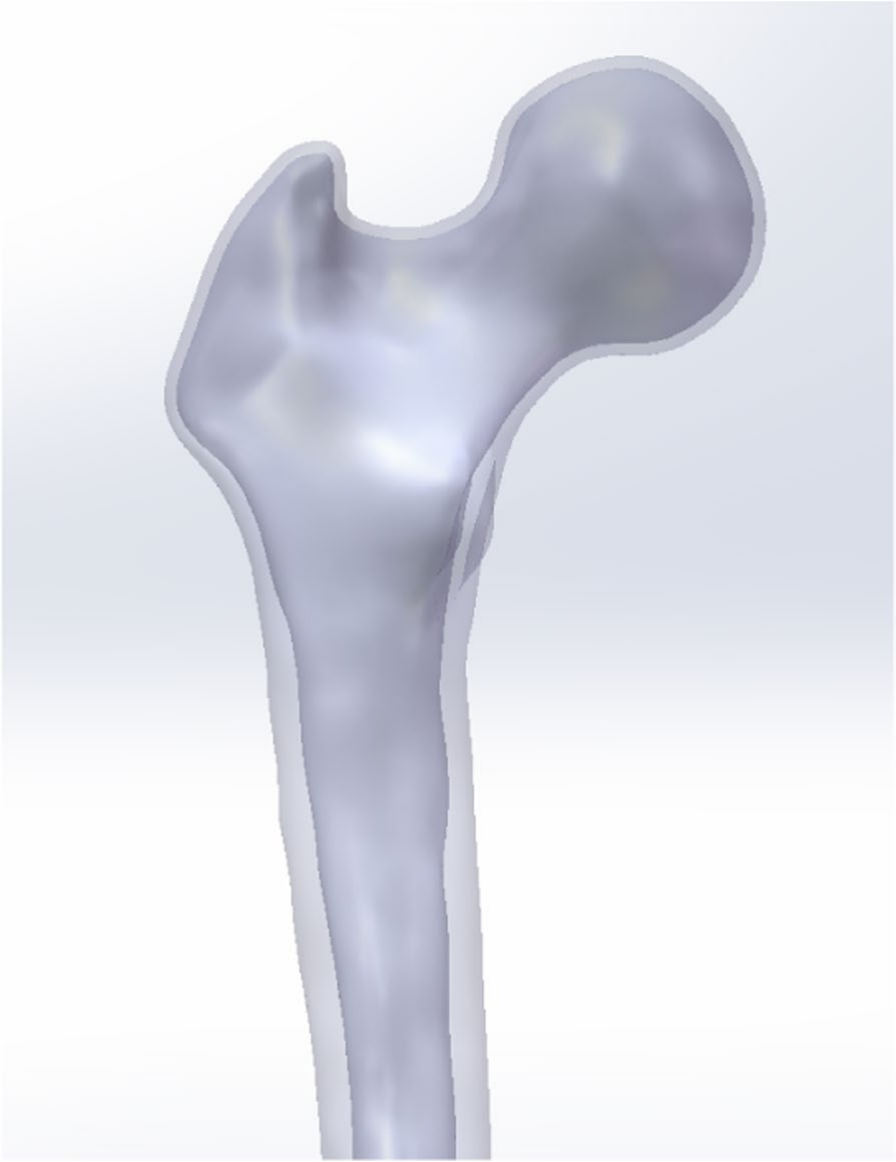
The three-dimensional model of the cortical bone and cancellous bone

Research involving human participants have been performed in accordance with the Declaration of Helsinki and have been approved by the Ethics Committee of the Second Affiliated Hospital of Guangzhou University of Chinese Medicine with the ethical registration ID YE2020-245. Femur computed tomography (CT) data were obtained from a 26-year-old male object using a Siemens 64-row CT scanner with a layer thickness of 0.7 mm were obtained. The CT image was stored in DICOM format and was outputted to the three-dimensional reconstruction medical software Mimics 21.0 (Materialise, Belgium) [17]. A three-dimensional model of the femur was built on the basis of the gray value of the tissue and segmentation of the region and then exported in stereolithography (STL) format. This STL format was imported into Geomagic Wrap 2017 software (Geomagic, USA) for smoothing, meshing, noise reduction and surface fitting; data were later imported into SolidWorks 2017 software (Dassault, France) [13, 18]. The three-dimensional model of the cortical bone and cancellous bone (Fig. 1) was developed by Boolean operations, and the proximal femoral bone model was built for reassembly [13].

**Fig. 2:**
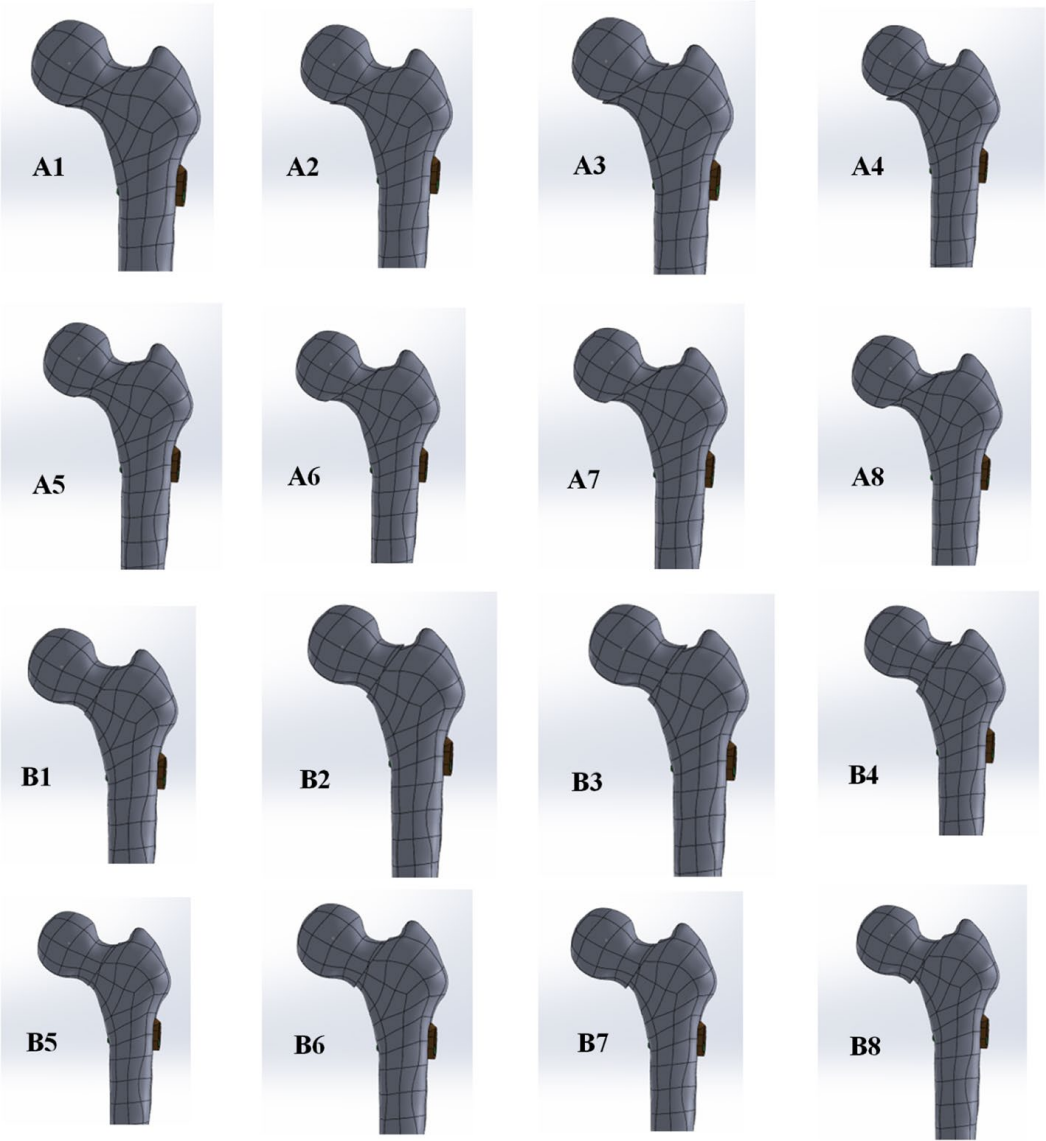
(A1) Positive 1-mm model, (A2) positive 2-mm model, (A3) positive 3-mm model, (A4) positive 4-mm model, (A5) negative 1-mm model, (A6) negative 2-mm model, (A7) negative 3-mm model, and (A8) negative 4-mm model at Pauwels angles of 30°; (B1) positive 1-mm model, (B2) positive 2-mm model, (B3) positive 3-mm model, (B4) positive 4-mm model, (B5) negative 1-mm model, (B6) negative 2-mm model, (B7) negative 3-mm model, and (B8) negative 4-mm model at Pauwels angles of 50°

Due to the angle between the horizontal line connecting the two iliac crests and the line of the distal segment of the fracture, femoral neck fractures were divided into the following three grades according to Pauwels classification: Type I, Pauwels angle < 30°; Type II, 30°~50°; and Type III, > 50° in 1935 [19]. Although the Pauwels classification was introduced decades ago, it is still classic and widely used in biomechanical evaluations [20, 21]. We constructed femoral neck fractures with Pauwels angles of 30° and 50° to stimulate subcephalic and transcervical femoral neck fractures. The proximal femoral bone model was developed using SolidWorks 2017 software (Dassault, France). The specific operations were as follows: to simulate three different angle fracture models, we first established a horizontal plane through the center of the femoral head, and then we drew a straight line near the neck of the femur; this straight line formed an angle of 30° or 50° with the horizontal line. It was divided into 1- to 4-mm displacement according to the distance between the positive buttress and negative buttress (Fig. 2).

Using SolidWorks 2017 software, we built FNS according to real clinical implant geometric data. In the construction of the FNS model, a sliding hip screw with a diameter of 10 mm was placed at an angle of 130° to the locking plate, and a locking anti-rotational screw with a diameter of 6.4 mm was placed at an angle of 7.5° to the sliding hip screw at the proximal end of FNS. At the distal end, a hole was made for a 5-mm locking screw.

**Fig. 3:**
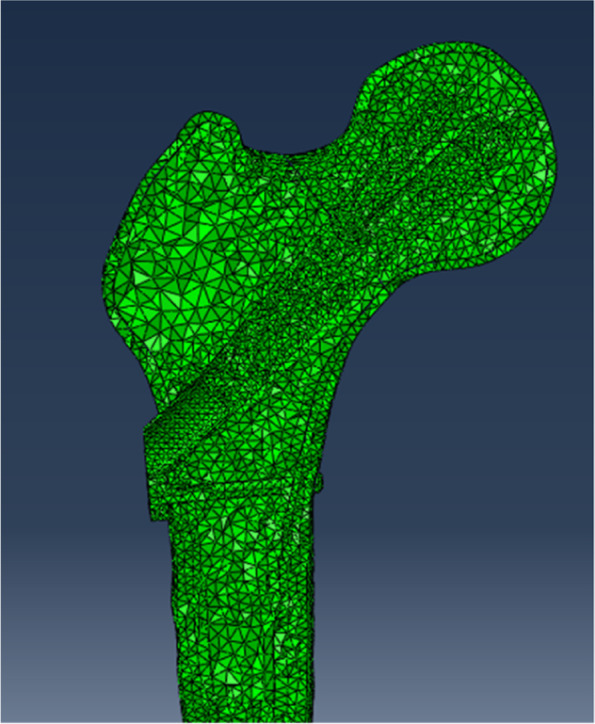
Cross-sectional view pictures of volume mesh of the femur(cortical bone and cancellous bone)and fixation

As the focus of this study was not related to the thread, threaded screw sections were modeled as smooth surfaces with diameters corresponding to the designed thread diameters to simplify the model. The plates and screws were composed of titanium alloy. The models were imported into Abaqus 2017 software (Simulia, France) for meshing (Fig. 3). Each assembly was meshed by tetrahedral 10-node elements (C3D10).

## Material parameters

For modeling purposes, it was assumed that the models comprised continuous, isotropic and uniform linear elastic materials. The number of nodes and elements of the four fixation models and the elastic modulus of the bones and implants are listed in Tables 1 and 2.

**Table 1:** Properties of the materials used in the present study (titanium alloy, cortical, and cancellous bone)

Titanium alloy		Cortical bone		Cancellous bone	
E (GPa)	Poisson’s ratio	E (GPa)	Poisson’s ratio	E (GPa)	Poisson’s ratio
105	0.35	16.8	0.3	0.84	0.2

**Table 2:** Details of the three assembly units and the total number of nodes

Case group	Pauwels angle of 30°	Pauwels angle of 50°
Anatomic reduction group		
Node	404,356	367,375
Unit	261,826	237,223
Mesh size	Maximum: 2 mm; minimum: 1.5 mm	
Positive buttress reduction group		
Node	356,984	359,218
Unit	229,967	231,851
Mesh size	Maximum: 2 mm; minimum: 1.5 mm	
Negative buttress reduction group		
Node	364,990	380,371
Unit	235,903	246,747
Mesh size	Maximum: 2 mm, minimum: 1.5 mm	

## Boundary conditions and loading force settings

**Fig. 4:**
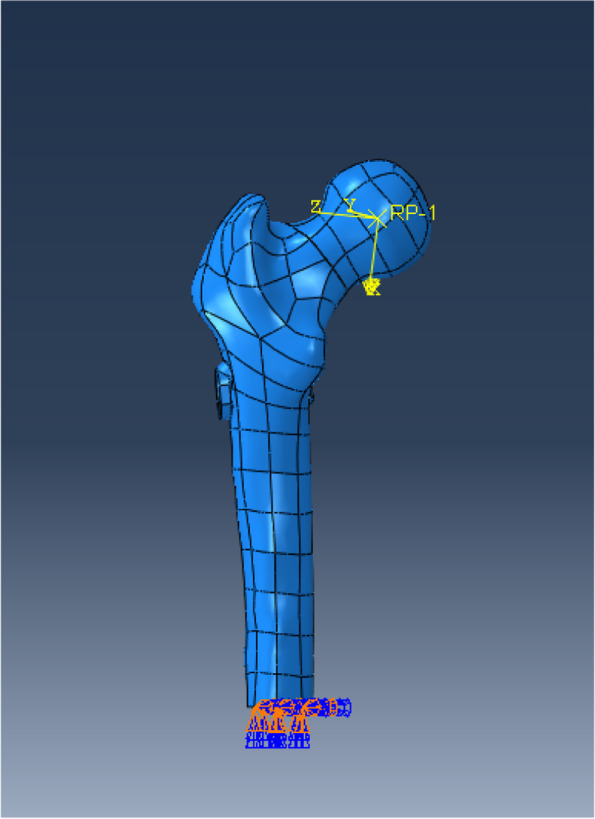
Loading (Arrow:2100 N) and boundary conditions of femoral model

For calculation purposes, the distal end of the femur was completely fixed (Fig. 4). According to the research by Van Houcke et al [22], the force of the standing joint of one leg was approximately 3.0 times the body weight. Therefore, in the finite element models, loads of 2100 N, equivalent to tripling the body weight of the subject, were applied to the center of the femoral head (Fig. 4). According to the setup previously introduced [23], we set the force vector pointing laterally at an angle of 13° to the axis of the femoral shaft in the coronal plane and posteriorly at an angle of 8° to the shaft in the sagittal plane.

## Contact settings

According to the well-established and approved test contact setup method described in previous studies [24–26], binding contact was formed between the internal fixation screw and the femur. Friction contact was used on the fracture surface with a friction coefficient of 0.46.

## Evaluation criteria

In finite element analysis, the displacements and stress of the femur and internal fixations were measured in each group. In addition, variation in each parameter was assessed in each group.

## Results

### Von Mises stress (VMS) of FNS internal fixation components

**Fig. 5:**
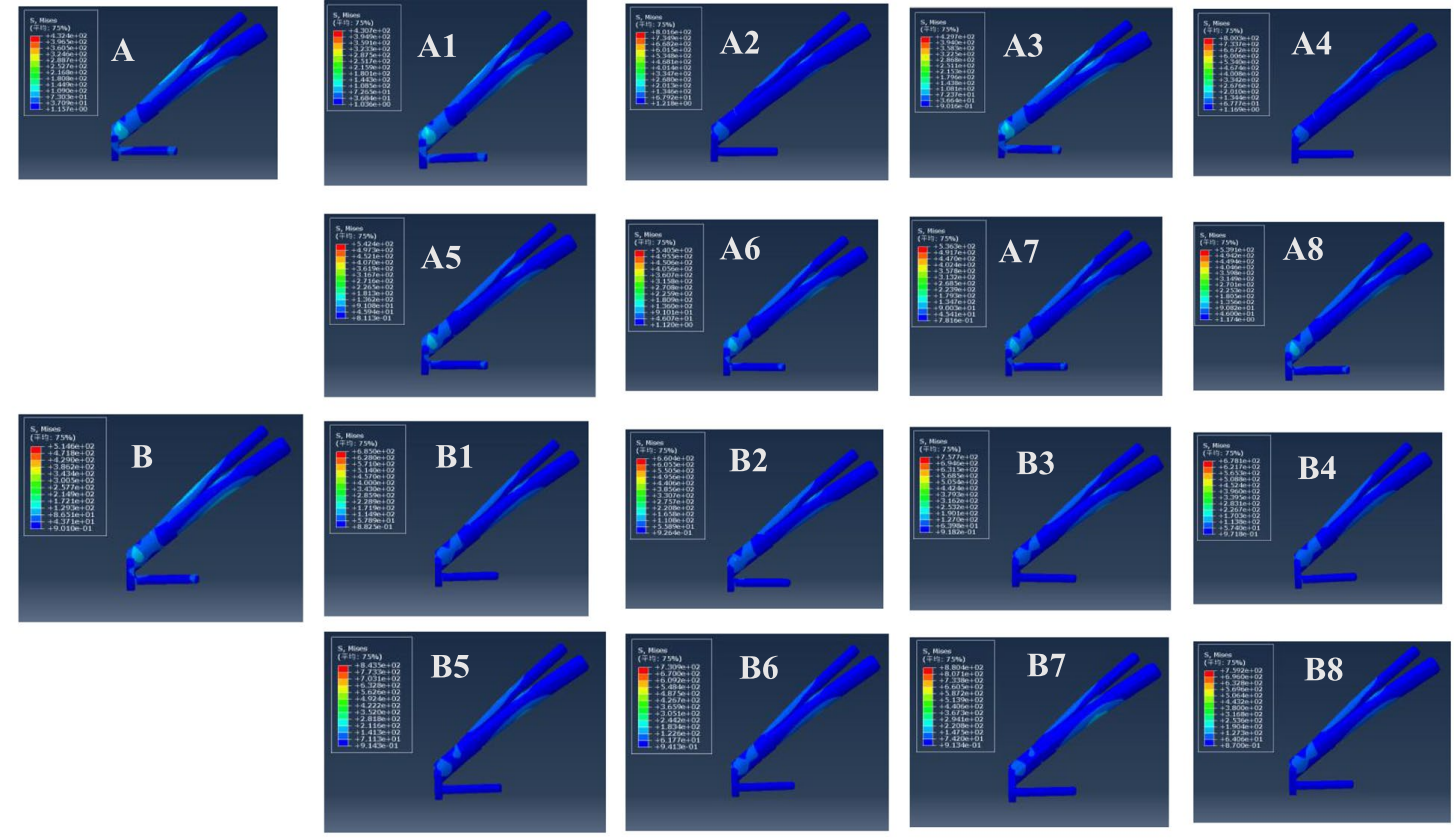
Maximum stress of FNS internal fixation. **A** Anatomic reduction model, (A1) positive 1-mm model, (A2) negative 1-mm model, (A3) positive 2-mm model, (A4) negative 2-mm model, (A5) positive 3-mm model, (A6) negative 3-mm model, (A7) positive 4-mm model, and (A8) negative 4-mm model at Pauwels angles of 30°; **B** anatomic reduction model, (B1) positive 1-mm model, (B2) negative 1-mm model, (B3) positive 2-mm model, (B4) negative 2-mm model, (B5) positive 3-mm model, (B6) negative 3-mm model, (B7) positive 4-mm model, (B8) and negative 4-mm model at Pauwels angles of 50°

**Fig. 6:**
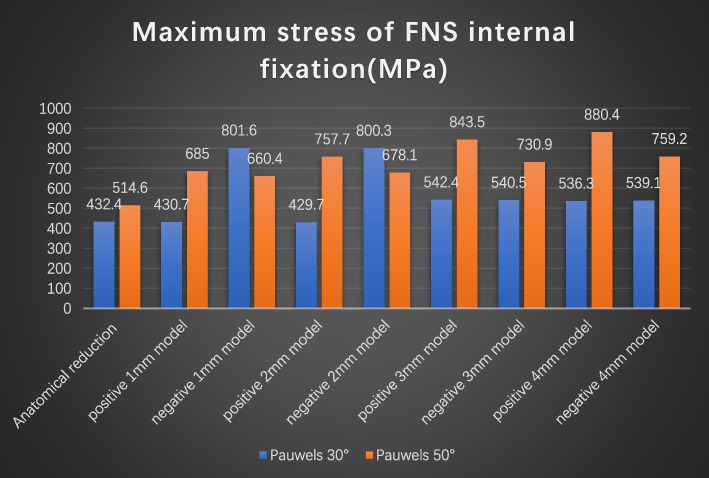
Graphic demonstration of the maximum stress of FNS internal fixation at Pauwels angles of 30° and 50°

The VMS distributions for positive buttress and negative buttress of FNS with Pauwels angles of 30° and 50° were assessed, as shown in Figs. 5 and 6. The stresses appeared to be concentrated at the junction of the sliding hip screw and anti-rotational screw and were distributed evenly along the screw. At a Pauwels angle of 30°, the peak VMS values of FNS were 432.4 MPa for the anatomic reduction model, 430.7 MPa for the positive 1-mm model, 801.6 MPa for the negative 1-mm model, 429.7 MPa for the positive 2-mm model, 800.3 MPa for the negative 2-mm model, 542.4 MPa for the positive 3-mm model, 540.5 MPa for the negative 3-mm model, 536.3 MPa for the positive 4-mm model, and 539.1 MPa for the negative 4-mm model. At a Pauwels angle of 50°, the peak VMS values of the FNS were 514.6 MPa for the anatomic reduction model, 685 MPa for the positive 1-mm model, 660.4 MPa for the negative 1-mm model, 757.7 MPa for the positive 2-mm model, 678.1 MPa for the negative 2-mm model, 843.5 MPa for the positive 3-mm model, 730.9 MPa for the negative 3-mm model, 880.4 MPa for the positive 4-mm model, and 759.2 MPa for the negative 4-mm model.

## Von Mises stress (VMS) of the femur

**Fig. 7:**
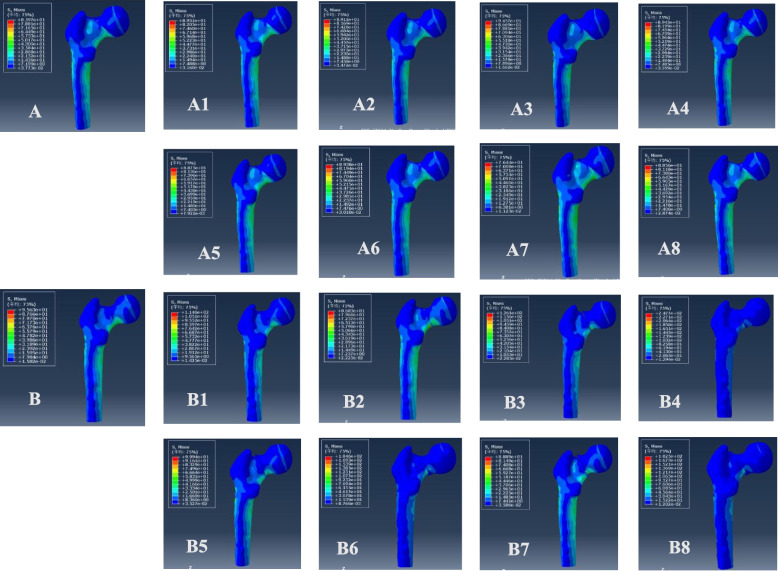
Maximum stress of femur. **A** Anatomic reduction model, (A1) positive 1-mm model, (A2) negative 1-mm model, (A3) positive 2-mm model, (A4) negative 2-mm model, (A5) positive 3-mm model, (A6) negative 3-mm model, (A7) positive 4-mm model, and (A8) negative 8-mm model at Pauwels angles of 30°; **B** anatomic reduction model, (B1) positive 1-mm model, (B2) negative 1-mm model, (B3) positive 2-mm model, (B4) negative 2-mm model, (B5) positive 3-mm model, (B6) negative 3-mm model, (B7) positive 4-mm model, and (B8) negative 4-mm model at Pauwels angles of 50°

**Fig. 8:**
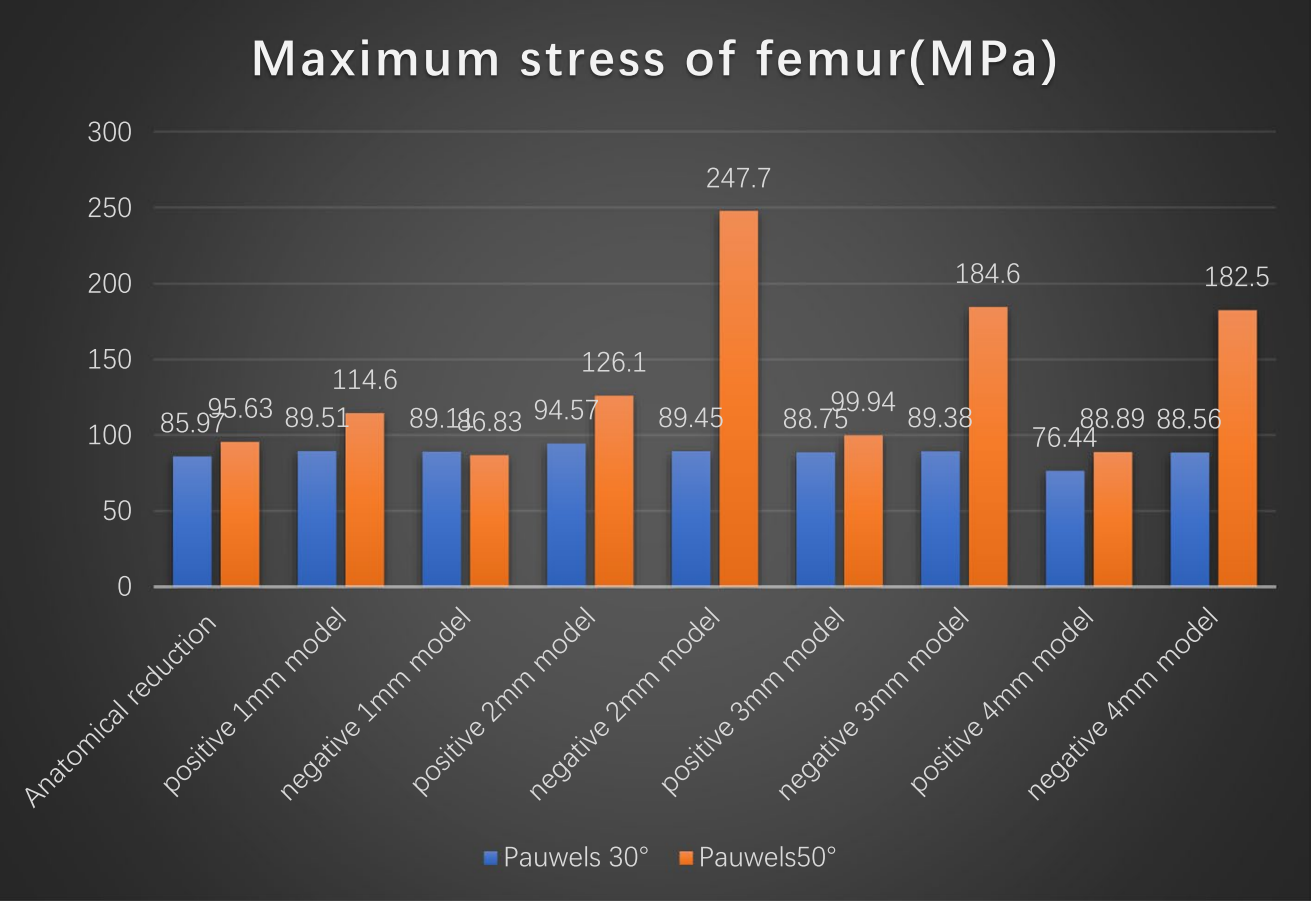
Graphic demonstration of the maximum stress of the femur at Pauwels angles of 30° and 50°

The VMS distributions for positive buttress and negative buttress of femoral neck fracture with Pauwels angles of 30° and 50° were also evaluated (Figs. 7 and 8). The maximum stress was sustained on the femoral calcar. The peak VMS values of the femur were 85.97 MPa for the anatomic reduction model, 89.51 MPa for the positive 1-mm model, 89.11 MPa for the negative 1-mm model, 94.57 MPa for the positive 2-mm model, 89.45 MPa for the negative 2-mm model, 88.75 MPa for the positive 3-mm model, 89.38 MPa for the negative 3-mm model, 76.44 MPa for the positive 4-mm model and 88.56 MPa for the negative 4-mm model at a Pauwels angle of 30°. At a Pauwels angle of 50°, these peak values were 95.63 MPa, 114.6 MPa, 86.83 MPa, 126.1 MPa, 247.7 MPa, 99.94 MPa, 184.6 MPa, 88.89 MPa, and 182.5 MPa, respectively.

## Displacement of FNS internal fixation components

**Fig. 9:**
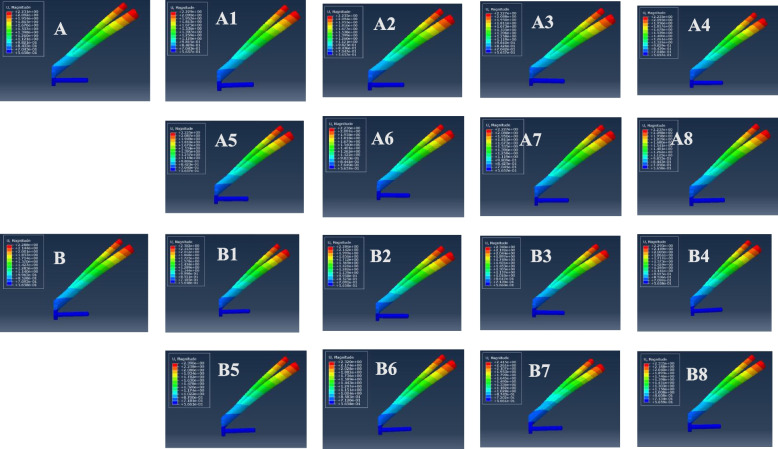
Maximum displacement of FNS internal fixation. **A** Anatomic reduction model, (A1) positive 1-mm model, (A2) negative 1 -mm model, (A3) positive 2-mm model, (A4) negative 2-mm model, (A5) positive 3-mm model, (A6) negative 3-mm model, (A7) positive 4-mm model, and (A8) negative 8-mm model at Pauwels angles of 30°; **B** anatomic reduction model, (B1) positive 1-mm model, (B2) negative 1-mm model, (B3) positive 2-mm model, (B4) negative 2-mm model, (B5) positive 3-mm model, (B6) negative -mm model, (B7) positive 4-mm model, and (B8) negative 4-mm model at Pauwels angles of 50°

**Fig. 10:**
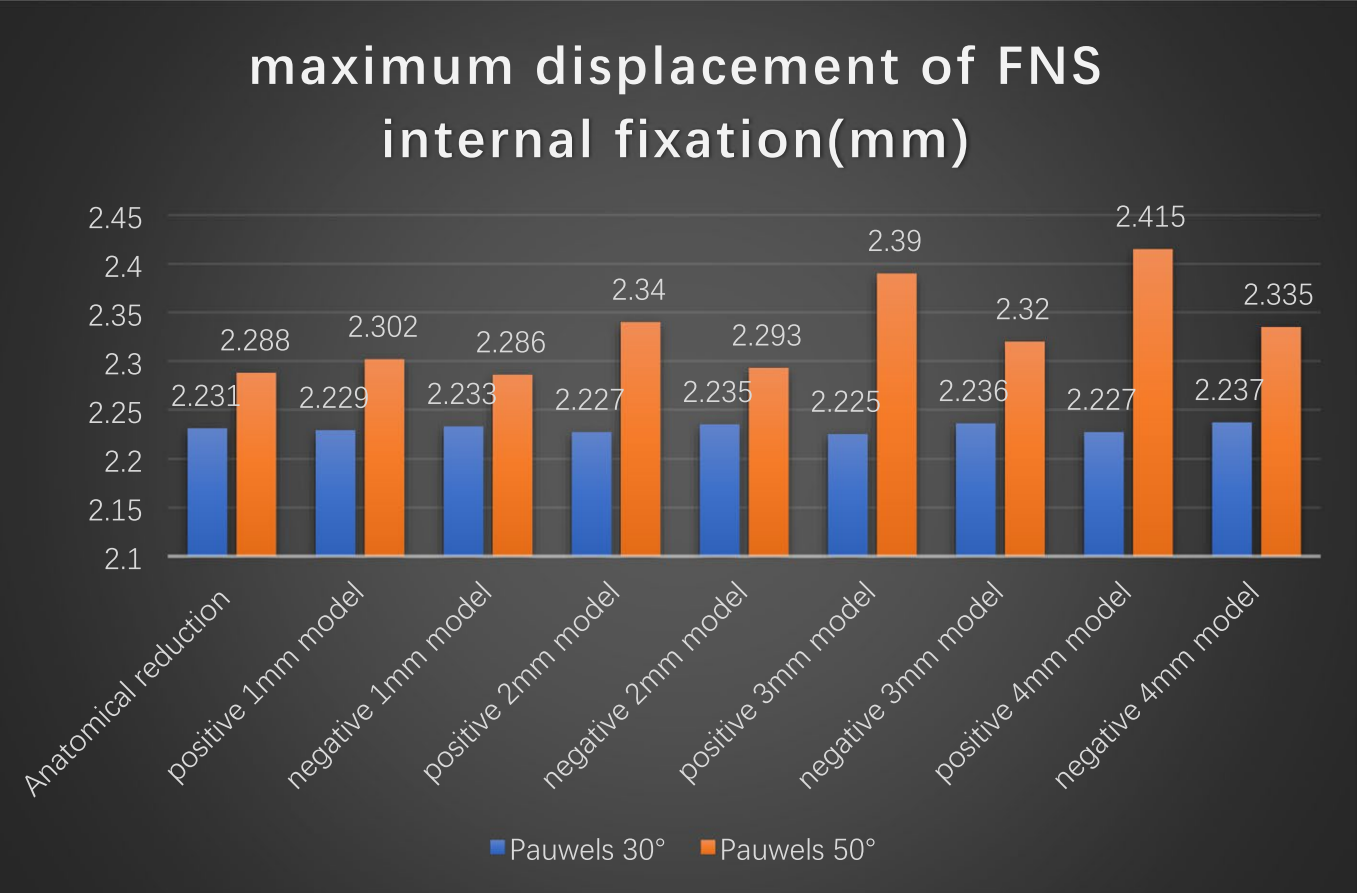
Graphic demonstration of the maximum displacement of FNS internal fixation at Pauwels angles of 30° and 50°

The maximum displacement of internal fixation occurred at the screw tail, depicted in Figs. 9 and 10. The maximum FNS displacements were 2.231 mm for the anatomic reduction model, 2.229 mm for the positive 1-mm model, 2.233 mm for the negative 1-mm model, 2.227 mm for the positive 2-mm model, 2.235 mm for the negative 2-mm model, 2.225 mm for the positive 3-mm model, 2.236 mm for the negative 3-mm model, 2.227 mm for the positive 4-mm model, and 2.237 mm for the negative 4-mm model at a Pauwels angle of 30°. These values at a Pauwels angle of 50° were 2.288 mm, 2.302 mm, 2.286 mm, 2.340 mm, 2.293 mm, 2.390 mm, 2.320 mm, 2.415 mm, and 2.335 mm, respectively.

## Displacement of the femur

**Fig. 11:**
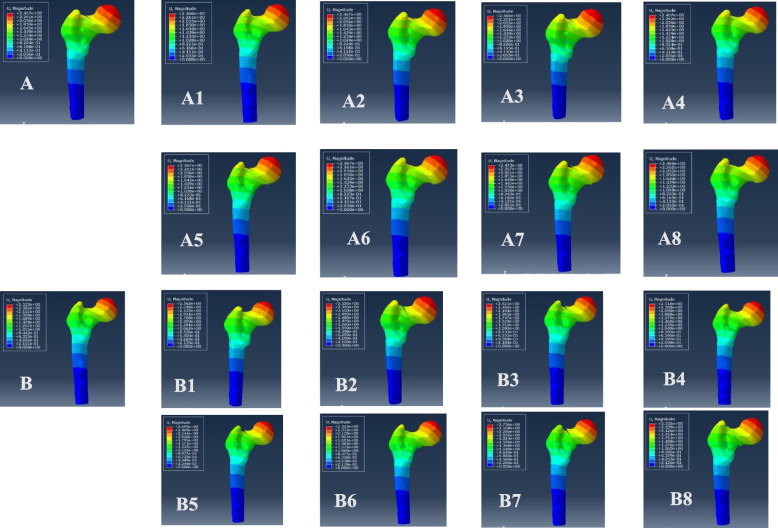
Maximum displacement of the femur. **A** Anatomic reduction model, (A1) positive 1-mm model, (A2) negative 1 -mm model, (A3) positive 2-mm model, (A4) negative 2-mm model, (A5) positive 3-mm model, (A6) negative 3-mm model, (A7) positive 4-mm model, and (A8) negative 8-mm model at Pauwels angles of 30°; **B** anatomic reduction model, (B1) positive 1-mm model, (B2) negative 1-mm model, (B3) positive 2-mm model, (B4) negative 2-mm model, (B5) positive 3-mm model, (B6) negative -mm model, (B7) positive 4-mm model, and (B8) negative 4-mm model at Pauwels angles of 50

**Fig. 12:**
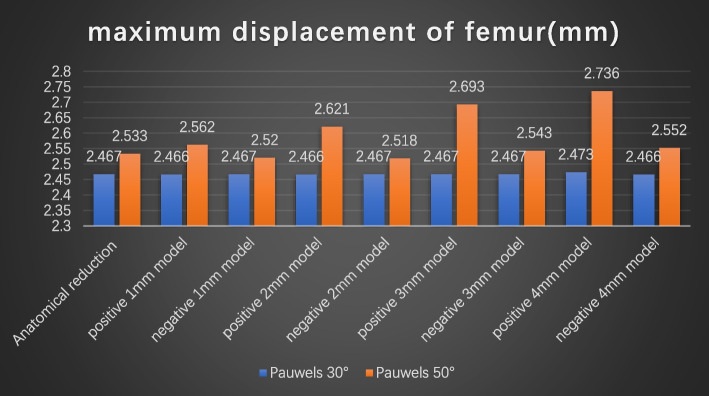
Graphic demonstration of the maximum displacement of the femur at Pauwels angles of 30° and 50

According to the displacement contours of the femur with Pauwels fracture at angles of 30° and 50°, the maximum displacement occurred at the upper part of the femoral head, as shown in Figs. 11 and 12. The displacements of the femur were 2.467 mm for the anatomic reduction model, 2.466 mm for the positive 1-mm model, 2.467 mm for the negative 1-mm model, 2.466 mm for the positive 2-mm model, 2.467 mm for the negative 2-mm model, 2.467 mm for the positive 3-mm model, 2.467 mm for the negative 3-mm model, 2.473 mm for the positive 4-mm model, and 2.466 mm for the negative 4-mm model at a Pauwels angle of 30°. The displacements of the proximal femur were 2.533 mm for the anatomic reduction model, 2.562 mm for the positive 1-mm model, 2.520 mm for the negative 1-mm model, 2.621 mm for the positive 2-mm model, 2.518 mm for the negative 2-mm model, 2.693 mm for the positive 3-mm model, 2.543 mm for the negative 3-mm model, 2.736 mm for the positive 4-mm model, and 2.552 mm for the negative 4-mm model at a Pauwels angle of 50°.

## Discussion

In our study, we explored the biomechanical outcomes of positive buttress and negative buttress of FNS internal fixation in the treatment of nonanatomically reduced femoral neck fracture based on finite element analysis. When the Pauwels angle was 30°, the positive 1-mm and 2-mm models had lower FNS stress than the negative buttress model. The positive 3- and 4-mm models showed FNS stress similar to that of the negative buttress model. But the four positive buttress models had similar stresses on the femur as the negative buttress model. When the Pauwels angle was 50°, the four positive buttress models had higher FNS stress than the negative buttress model, and the three positive buttress models (2 mm, 3 and 4 mm) displayed lower stress for the femur than the negative buttress model, which was not observed for the 1-mm model. Hence, positive and negative buttress in the treatment of femoral neck fracture with FNS will vary due to the Pauwels angle. When the fracture angle was 30°, the positive buttress group was superior to the negative buttress in terms of FNS stress, and the two groups were basically equal in terms of femoral stress. When the fracture angle was 50°, FNS internal implant bear more stress in the positive buttress group than negative buttress, resulting in less femoral stress in the positive buttress.

When the Pauwels angle was 30°, the positive buttress model had a lower displacement of the FNS than the negative buttress model, but the displacement of the femur similar to that of the negative buttress model. When the Pauwels angle was 50°, the positive buttress model had a higher displacement of both FNS and femur than the negative buttress model. This means that the positive buttress group was more stable than the negative buttress at a Pauwels angle of 30° but may not at a Pauwels angle of 50°.

Traditionally, “anatomical reduction” is a key factor in promoting fracture healing and avoiding postoperative complications [14], which has never been questioned. The real problem is that regardless of effort, there is still a high possibility of encountering a refractory femoral neck fracture, and it is difficult to achieve anatomical reduction under closed reduction in such cases. Therefore, we explored how to perform FNS internal fixation for femoral neck fractures in young patients without anatomic reduction. The concept of Gotfried reduction for femoral neck fracture has been proposed for almost a decade. Several studies have shown that Gotfried positive buttress reduction and fixation for femoral neck fracture result in similar clinical effects with anatomic reduction but are much better than Gotfried negative buttress reduction [27, 28].

The technique of Gotfried reduction is to stabilize unstable sub-cephalic fractures [29]. In our study, Pauwels type I and type II femoral neck fractures were adopted as the fracture mode. Our results show that when the Pauwels angle was 30°, positive buttress was superior to negative buttress. However, when the Pauwels angle was 50°, this advantage will weaken. We also observed this with femoral displacement: when the angle was 30°, the effect of the positive buttress was more stable than negative buttress; this advantage is not seen in the case at Pauwels angles of 50°. As the Pauwels angle increased, the Von Mises stress and displacement of FNS fixation and the femur also increased. A retrospective clinical study from Zhao et al [27] found that positive buttress position reduction of femoral neck fractures in young patients showed a lower incidence of complications and reoperations compared with those of negative reduction using three parallel cannulated screws. Another retrospective study found that anatomic reduction and Gotfried positive buttress reduction group had higher Harris hip scores and lower femoral neck shortening than Gotfried negative buttress and suggested that achieving positive valgus reduction can also obtain satisfactory clinical results and should try to avoid negative buttress [28]. Our findings are partial consistent with previous studies [16, 27, 28, 30], which reported that positive buttress is better than negative buttress. Possible explanations may be related to the following aspects. First, the Gotfried reduction method was first applied to sub-cephalic femoral neck fractures. In our study, when the Pauwels angle was 30°, it was considered a sub-cephalic fracture, consistent with the results of previous studies. When the Pauwels angle was 50°, it was considered a transcervical femoral neck fracture, which may be the source of the inconsistency. Second, when Gotfried et al. presented their concept, they established three pre-requisitives for sub capital fractures to heal: a positive buttress reduction, minimum neck-shaft angle of 135 degrees, and 180 degrees alignment in the lateral view or a minimum of 160 degrees. Our model only satisfies positive buttress reduction and does not incorporate two out of three major parameters. Therefore, our findings can only explain the stability of FNS in femoral neck fractures under non-anatomical reduction, but not under Gotfried positive support concept. Third, our model assumed movement on a smooth fracture surface, rather than interlocking the fracture ends, as in the original Gotfried reduction. Finally, all previous clinical studies used three cannulated screws for internal fixation, which is different from our FNS internal fixation, and the difference in internal fixation type is also one of the reasons for the inconsistent results. In our opinion, the clinical efficacy of FNS internal fixation with positive buttress may be related to the fracture angle, neck-shaft angle and alignment in the lateral view.

The limitations of our study are similar to those inherent to all finite element studies, whereby the model in this study was based on the femur being set as a homogeneous, continuous and isotropic elastic material. However, human bone is an isotropic heterogeneous material; thus, the material properties in the finite element experiment may have affected the results. Moreover, the model does not reflect the real relationships between bone fragments which are observed in real fracture site. Smooth ends of bone fragments in the model which are not observed in reality. However, as an initial biomechanics report, it can be considered reasonable. In the future, we need to construct more realistic bone fragments in real fracture site. In addition, our binding contact is placed at the junction of the internal fixation and the bone. However, under the loading force, a relative movement occurs between the bone and the FNS locking plate. But our contact settings is based on previous literature. It was acceptable since we recreated the optimum state of stable contact between bone and internal fixation. Finally, our results have not been verified by animal or clinical experiments. Our research setting is effective because it is based on the previous verified research [13]. Nonetheless, our objective was to explore trends rather than absolute measurements. In this respect, the lack of experimental validation is rational.

## Conclusion

From the perspective of biomechanics, when the Pauwels angle was 30°, positive buttress was more stable to negative buttress. However, when the Pauwels angle was 50°, this advantage weakens. In our opinion, the clinical efficacy of FNS internal fixation with positive buttress may be related to the fracture angle, neck-shaft angle and alignment in the lateral view. This result needs verification in further clinical studies.

## Data Availability

The datasets used and/or analyzed during the current study are available from the corresponding author on reasonable request.
